# Tackling the Triple Threat in Kenya: Factors Associated with Protection against HIV Risk, Gender-Based Violence, and Pregnancy among Adolescent Girls and Young Women

**DOI:** 10.1007/s10461-025-04643-9

**Published:** 2025-02-13

**Authors:** Brendan Maughan-Brown, Boladé Hamed Banougnin, Madison T. Little, Lucas Hertzog, Ntombekhaya Matsha-Carpentier, Celestine Mugambi, Hermes Gichane, Lucie Cluver, Elona Toska

**Affiliations:** 1 Southern Africa Labour and Development Research Unit, University of Cape Town, Cape Town, 7700 South Africa; 2 United Nations Population Fund, West and Central Africa Regional Office, New York City, USA; 3 Department of Social Policy and Intervention, University of Oxford, Oxford, UK; 4 Curtin School of Population Health, Curtin University, Perth, Australia; 5 High Impact Africa 2 Department, The Global Fund to Fight AIDS, TB and Malaria, Geneva, Switzerland; 6 The National Syndemic Diseases Control Council (NSDCC), Nairobi, Kenya; 7 National AIDS and STI Control Program (NASCOP), Nairobi, Kenya; 8 Department of Psychiatry and Mental Health, University of Oxford, Oxford, UK; 9 Centre for Social Science Research, University of Cape Town, Cape Town, South Africa

**Keywords:** AGYW, Motherhood, Parenting practices, Gender norms, Food security, HIV, Kenya

## Abstract

The government of Kenya’s Triple Threat strategy recognises the need for urgent intervention to address the high incidence of HIV, gender-based violence (GBV) and pregnancy among adolescent girls and young women (AGYW). We used nationally representative data among AGYW aged 13–24 (*n* = 1344) from the 2018-19 Kenya Violence Against Children & Youth Survey (VACS) in multivariable logistic regression analyses to examine associations between three potential protective factors – gender-equitable attitudes, food security and parental monitoring – and outcomes related to the triple threat: Intimate partner violence (IPV); sexual violence; high HIV exposure risk; adolescent pregnancy; child marriage; and not being in school or paid work. Results showed that AGYW with gender-equitable attitudes were less likely to report IPV (adjusted odds-ratio (aOR):0.47, *p* < 0.01) and adolescent pregnancy (aOR:0.58, *p* < 0.05). AGYW who were food secure were less likely to report adolescent pregnancy (aOR:0.57, *p* < 0.05) and child marriage (aOR:0.51, *p* < 0.05). Those with high parental monitoring were less likely to report IPV (aOR:0.44, *p* < 0.01), sexual violence (aOR:0.49, *p* < 0.05), adolescent pregnancy (aOR:0.61, *p* < 0.05), and child marriage (aOR:0.41, *p* < 0.01). AGYW who had gender-equitable attitudes, food security *and* high parental monitoring (i.e., all three factors compared to none) had lower predicted probabilities of reporting IPV (22.3% vs. 5.0%), sexual violence (20.4% vs. 6.0%), adolescent pregnancy (23.7% vs. 7.7%), and child marriage (10.0% vs. 1.6%). The findings indicate that improving gender-equitable attitudes, food security and parental monitoring may positively impact multiple outcomes related to the triple threat; and that addressing all three may have the greatest impact.

**Supplementary Information:**

The online version contains supplementary material available at 10.1007/s10461-025-04643-9.

## Introduction

High incidence of HIV, gender-based violence (GBV), and pregnancy among adolescent girls and young women (AGYW) in Kenya all undermine individual well-being – profoundly impacting health, education, and economic opportunities – and are impediments to achieving the Sustainable Development Goals. These three outcomes, referred to as the ‘triple threat,’ are interconnected and require urgent intervention. In 2022, there were an estimated 5,720 new HIV infections among AGYW 15–24 years-old in Kenya [1]. Adolescent (10–19 years old) pregnancy – an outcome strongly associated with risk of exposure to HIV [2, 3] – was reported by 47% of 20–24 year-old women nationally in the Kenya DHS 2022, and by as many as 76% in counties with HIV incidence > 0.3% [4]. Sexual or physical violence – also known to increase risk of exposure to HIV [5] – was reported by 17% of AGYW in the past 12 months in the Kenya DHS 2022 [4]. The government of Kenya has called for a multisectoral response to the triple threat [6].

Structural, biomedical, socio-behavioural and psychological factors, individually and in combination, increase the likelihood of AGYW experiencing the triple threat [7]. Poverty, poor education outcomes, and child marriage are related to the triple threat as these factors increase the risk of HIV exposure and the likelihood of adolescent pregnancy through condomless sex, multiple sexual partners, age-disparate partnerships, and transactional sex [7–9]. The triple threat and its determinants are also influenced by harmful gender norms and related gender-inequitable attitudes [8, 10–12], and insufficient support from, or poor relationships with, parents [7, 13].

Evidence indicates that combination interventions, including changes in the broader environment which influence health behaviours, can be mutually reinforcing and result in greater benefits than stand-alone programmes and interventions [13–16]. To be most effective and targeted (given declining funding for the response against HIV), multi-component interventions are needed that impact multiple outcomes relating to the triple threat. Such interventions have been termed ‘development accelerators’: interventions, provisions, services, or programmatic areas that simultaneously make a positive impact across multiple outcomes in a given context for a specific population [17, 18].

While the range of interventions shown to impact specific AGYW outcomes is broad, the challenge is both to identify interventions that impact multiple outcomes, and to identify interventions with synergistic effects. To obtain insights into what interventions may be needed for AGYW, this study examines the association between three factors shown to improve multiple outcomes in other settings – gender-equitable attitudes [8, 10], parental monitoring or support [13, 14, 19–21], and food security [3, 13, 14, 20–22] – and high HIV exposure risk, experience of violence, and adolescent pregnancy among AGYW in Kenya.

## Methods

### Study Design and Population

A cross-sectional, nationally representative household survey of young people aged 13–24 years – the Violence Against Children & Youth Survey (VACS) – was conducted in Kenya between December 2018 and January 2019. The survey collected information on emotional, physical, and sexual violence; well-being; and sexual and reproductive health. The survey instruments included an individual questionnaire conducted with AGYW, and a household questionnaire conducted with the head of the household. The 2019 Kenya VACS used a three-stage stratified sample design. Enumeration areas were selected randomly at the first stage, households at the second stage, and an eligible participant from the household at the third stage. The Kenya VACS used a split sample approach, such that the survey for females was conducted in different areas from the survey for males. This approach was to protect the confidentiality of participants by eliminating the chance that perpetrators of violence would be interviewed in the same community as survivors, might discover the purpose of the study, and could possibly retaliate against participants [23, 24]. Parents or primary caregivers of participants aged 13–17 years, and participants aged 18–24 years, provided informed consent. Ethics approval for the Kenya VACS was provided by: the U.S. Centers for Disease Control and Prevention (CDC); University of California, San Francisco; University of Nairobi; and Population Council (protocol number 6538).

## Measures

### Independent Variables

We created three binary variables that we hypothesized would be associated with multiple health-related outcomes.

*Gender-equitable attitudes* (binary variable) indicated respondents who responded “no” to all 10 yes/no statements that measured support of inequitable gender norms and acceptance of intimate partner violence. Inequitable gender norms statements were: (1) “Only men, not women, should decide when to have sex”; (2) “If someone insults a boy or man, he should defend his reputation with force if he needs to”; (3) “There are times when a woman should be beaten”; (4) “Women who carry condoms have sex with a lot of men”; and (5) “A woman should tolerate violence to keep her family together.” These questions were adapted from the Gender Equitable Men (GEM) scale [25]. Acceptance of intimate partner violence was measured by asking: “In your opinion, is a husband justified in hitting or beating his wife in the following situations: 1. If she goes out without telling him; 2. If she neglects the children; 3. If she argues with him; 4. If she refuses to have sex with him; and 5. If she burns the food.” The 10 items used in the creation of our binary variable had high internal consistency (alpha: 0.80).

*Food security* identified individuals who responded “no” to the question “In the past month, was there a day that you went without food because there wasn’t enough food in the household?”.

Parental monitoring was measured using a five-item scale that has been used widely in the developmental literature to assess parents or caregivers having knowledge of participants’ friends, money management, whereabout after school or at night, and free-time management [26]. Specifically, the question asked: “How much does/did your father, mother or caregiver really know the following things – does/did he/she know a lot, a little, or nothing? 1. Who your friends are/were? 2. How you spend/spent your money? 3. Where you go/went after school? 4. Where you go/went at night? 5. What you do/did with your free time?” Present tense was used for respondents 13–17 years old and past tense for respondents 18–24 years old. The five items had high internal consistency (alpha: 0.82). Our binary independent variable – *high parental monitoring* – identified participants who reported ‘a lot’ for each question.

### Outcome Variables Operationalising the Triple Threat for AGYW in Kenya

*Intimate partner violence (IPV)* was defined as self-report of any emotional, physical or sexual violence perpetrated by an intimate partner in the past twelve months. Emotional IPV was measured by asking respondents whether a boyfriend/romantic partner, ex-boyfriend/romantic partner, husband or ex-husband had done any of these things in the past 12 months: (1) Insulted, humiliated, or made fun of you in front of others? (2) Kept you from having your own money? (3) Tried to keep you from seeing or talking to your family or friends? (4) Kept track of you by demanding to know where you were and what you were doing? (5) Made threats to physically harm you? Physical IPV was measured by asking whether a boyfriend/romantic partner, ex-boyfriend/romantic partner, husband or ex-husband had done any of these in the past 12 months: (1) Slapped, pushed, shoved, shaken, or intentionally thrown something at you to hurt you? (2) Punched, kicked, whipped, or beaten you with an object? (3) Choked, suffocated, tried to drown you, or burned you intentionally? (4) Used or threatened you with a knife, gun or other weapon? Sexual IPV was measured by asking whether a boyfriend/romantic partner, ex-boyfriend/romantic partner, husband or ex-husband had done any of these in the past 12 months: (1) Touched you in a sexual way without your permission, but did not try and force you to have sex? (2) Tried to make you have sex against your will but did not succeed? (3) Physically forced you to have sex and did succeed? (4) Pressured you to have sex, through harassment or threats and did succeed?

*Sexual violence*: VACS Kenya assessed four forms of sexual violence, as categorised in the VACS questionnaire: unwanted sexual touching (“Has [____] ever touched you in a sexual way without your permission, but did not try and force you to have sex?”); attempted forced sex (“Has [___] ever tried to make you have sex against your will but did not succeed?”); physically forced sex (“Has [___] ever physically forced you to have sex and did succeed?); and pressured sex (“Has [___] ever pressured you to have sex, through harassment or threats and did succeed?”). A binary variable was created to identify respondents who reported one or more of these forms of sexual violence by anyone in the past 12 months.

*High HIV exposure risk* was defined as self-reported condomless sex in combination with any of the following in the past 12 months: (1) sex with multiple partners; (2) sex with partners five or more years older; or (3) sex in exchange for money or any material support (transactional sex). The binary outcome variable was based on information provided about respondents’ three most recent partners in the past 12 months. Survey participants were provided the following definition of sex: “‘sex’ refers to anytime a man’s penis enters your vagina, or your anus, or your mouth, however slight, or the insertion of an object into your vagina or anus by someone else.”

*Adolescent pregnancy* was measured as self-report of ever being pregnant before the age of 20. Data were not available to determine whether pregnancies were wanted or unwanted.

*Child marriage* was defined as self-report of being married or living with a partner as if married prior to the age of 18.

*Not in school or paid work*. Not in school or paid work was defined as neither being enrolled in school at the time of the survey nor having had a paid job in the twelve months prior to the survey.

### Covariates

Covariates included: participant’s age (in years); absent parent (defined as reporting either that at least one parent was deceased, or not knowing whether they had died); place of residence (urban vs. rural); and household poverty (defined as the two lowest wealth quintiles obtained from principal component analysis on household assets and housing characteristics). In addition, we controlled for secondary school completion, as poorer education outcomes, including failure to complete secondary school, have been associated with HIV incidence and pregnancy [27–30]. Data on whether respondents were living with their parents were not collected, and this was therefore not included as a control variable.

### Statistical Analysis

The VACS complex survey design was accounted for using survey weights and adjusting variance estimates with the Taylor series linearisation [31]. Descriptive statistics (weighted proportions) were calculated for demographic characteristics, hypothesised accelerators, factors associated with high HIV exposure risk, and violence-related outcomes. Multivariable logistic regression, adjusting for potential covariates, assessed the association between hypothesised accelerators and the outcome variables. Westfall-Young stepdown adjusted p-values with 10 000 bootstrap replications were used to reduce the likelihood of false rejections [32–34]. A sensitivity analysis was conducted using the scale measure for gender-equitable attitudes and parental monitoring to examine whether the selection of the cut-off for our main binary outcomes had influenced the results. Finally, predicted probabilities of experiencing each outcome were computed for the combination of all three accelerators (gender-equitable attitudes, food-security and high parental monitoring) vs. none of the three accelerators. There was a small amount of missing data (see Table 1). Observations with missing data were excluded from the analysis. All analyses were done using Stata 18.

**Table 1:** Sample characteristics among AGYW 13–24 years old

	*n*/*N*	Missing data (*n*)	Weighted % (95% CI)
**Covariates**			
Rural residence	828/1344	0	65.2 (57.1–72.5)
Age group		0	.
13–17	653/1344		44.9 (41.0–48.9)
18–24	691/1344		55.1 (51.1–59.0)
Absent parent	343/1344	0	25.2 (21.7–29.0)
Lived in poor household	579/1344	0	41.2 (34.8–47.9)
Secondary school completed	237/1344	0	20.4 (17.1–24.0)
**Hypothesized accelerators**			
Gender equitable attitudes	398/1344	0	32.5 (28.3–37.0)
Food security	1005/1339	5	76.0 (71.9–79.6)
High parental monitoring	359/1313	31	28.1 (24.0–32.7)
**Outcomes**			
Intimate partner violence	203/1316	28	15.2 (12.5–18.3)
Sexual violence	173/1344	0	14.0 (11.3–17.2)
High HIV exposure risk	157/1344	0	10.9 (8.9–13.2)
Adolescent pregnancy	231/1335	9	15.7 (13.4–18.5)
Not in school or paid work	342/1344	0	24.9 (21.4–28.8)
Child marriage before age 18	95/1312	32	5.3 (3.9–7.1)

## Results

The overall response rate for females was 74.0%, with a 90.5% response rate at the household level, and 81.7% for individuals. A total of 1344 AGYW 13–24 years old were included in the study. Table 1 shows that two thirds of AGYW were living in rural areas at the time of the survey. Slightly more AGYW were 18 and older, with a mean age of 18.1. Almost one in four had an absent parent. In terms of the potential accelerators, three quarters of AGYW (76%) were food secure, one quarter (28%) reported high parental monitoring, and one third (32.5%) reported gender-equitable attitudes. Outcome data show that 15% of adolescents reported IPV and 14% reported sexual violence. 11% of AGYW self-reported behaviours that we categorised as high HIV exposure risk. Adolescent pregnancy was reported by 16% of all AGYW, and child marriage by 5%. One quarter of AGYW were out of school or not in paid work. Among the sub-sample aged 20 years and older the prevalence of adolescent pregnancy was 33%, and among the sub-sample aged 18 years and older the prevalence of child marriage was 9.5% (not shown in Table 1).

**Fig. 1:**
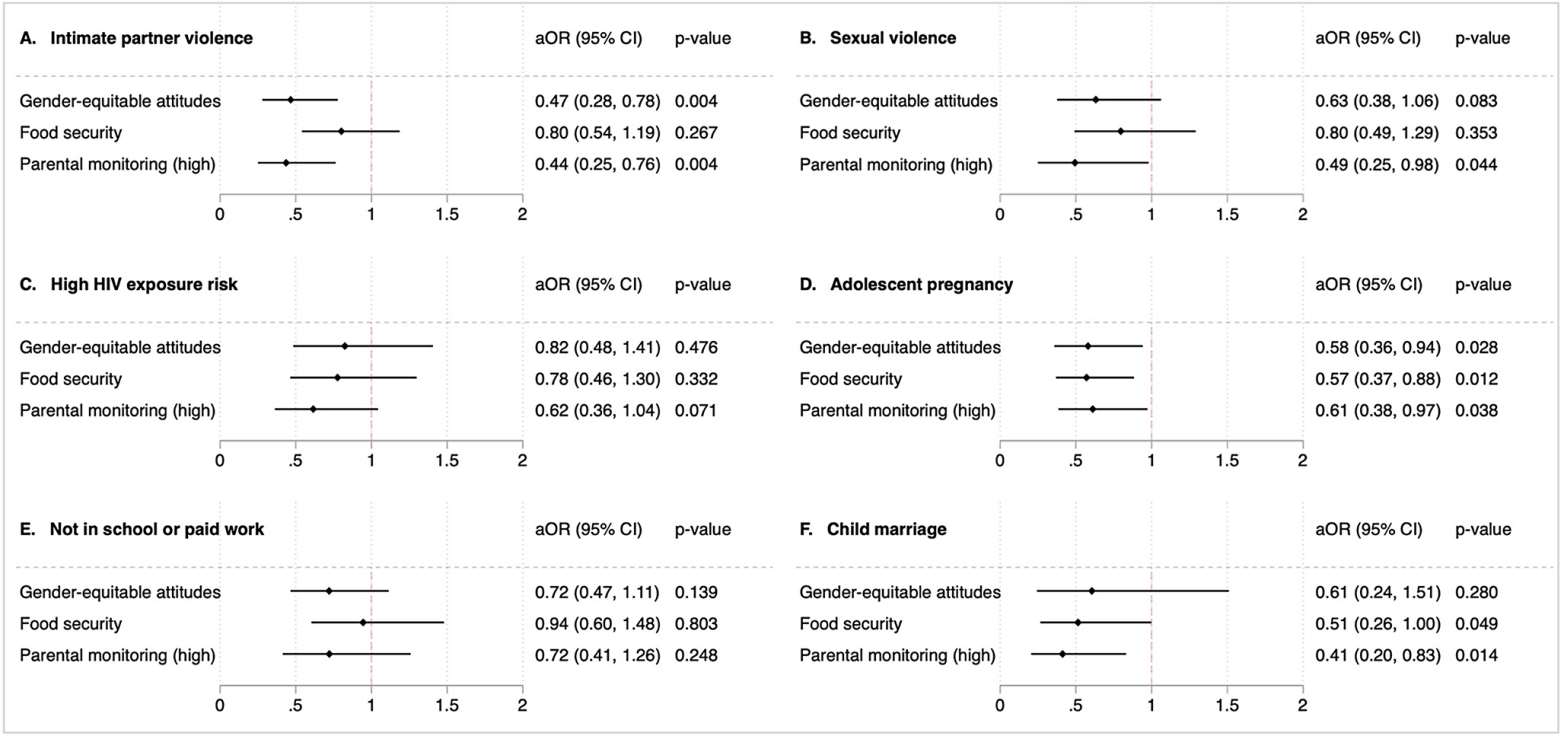
Associations between hypothesised accelerators and individual outcomes: **A**. Intimate partner violence; **B**. Sexual violence; **C**. High HIV exposure risk; **D**. Adolescent pregnancy; **E**. Not in school or paid work; **F**. Child marriage. Adjusted odds ratios (aOR) and 95% confidence intervals (CI) are adjusted for rural (vs. urban) residence, age, household poverty, absent parent, and secondary school completion

All three hypothesised accelerators were associated with statistically significant decreased probabilities of reporting at least two HIV risk factors or violence outcomes (see Fig. 1). Having gender-equitable attitudes was associated with lower probabilities of reporting IPV (adjusted odds ratios [aOR]: 0.47, 95% CI: 0.28–0.78, *p* < 0.01) and adolescent pregnancy (aOR: 0.58, 95% CI: 0.36–0.94, *p* < 0.05). Participants who were food-secure were less likely to report adolescent pregnancy (aOR: 0.57, 95% CI 0.37–0.88, *p* < 0.05) and child marriage (aOR: 0.51, 95% CI: 0.26–1.00, *p* < 0.05) than those who were food insecure. Respondents who reported high parental monitoring were less likely to report IPV (aOR: 0.44, 95% CI: 0.25–0.76, *p* < 0.01), sexual violence (aOR: 0.49, 95% CI: 0.25–0.98, *p* < 0.05), adolescent pregnancy (aOR: 0.61, 95% CI: 0.38–0.97, *p* < 0.05), and child marriage (aOR: 0.41, 95% CI: 0.20–0.83, *p* < 0.01). See Table S1, Supplemental Material File, for full regression results.

Sensitivity analysis found that results were robust in models that included the scale measures for gender-equitable attitudes (0–10) and parental monitoring (0–15) – see Supplemental Material File, Figure S1. In these modes, in addition to the main findings, a negative relationship was also found between gender-equitable attitudes and not being in school or paid work (aOR: 0.86, 95% CI: 0.80–0.93, *p* < 0.01), and child marriage (aOR: 0.87, 95% CI: 0.76–0.99, *p* < 0.05). In addition to the main findings, a negative relationship was also found between parental monitoring and high HIV exposure risk (aOR: 0.89, 95% CI: 0.84–0.95, *p* < 0.01), and not being in school or paid work (aOR: 0.91, 95% CI: 0.85–0.98, *p* < 0.05).

Predicted probabilities for individual outcomes associated with reporting all three accelerators versus none are presented in Table 2. Exposure to all three accelerators (vs. none) was associated with significant decreased probability of experiencing IPV (22.3% vs. 5.0%, risk difference (RD): -17.2%-points, 95% CI: -24.3 to -10.2), sexual violence (20.4% vs. 6.0%, RD: -14.4%-points, 95% CI: -24.2 to -4.6), adolescent pregnancy (23.7% vs. 7.7%, RD: -16.0%-points, 95% CI: -23.5 to -8.4), and child marriage (10.0% vs. 1.6%, RD: -8.4%-points, 95% CI: -12.9 to -4.0). Exposure to all three accelerators (vs. none) had a substantially larger risk difference compared to the individual accelerators.

**Table 2:** Covariate-adjusted predicted probabilities and risk differences for outcomes associated with exposure to accelerators

	Intimate partner violence	Sexual violence	High HIV exposure risk	Adolescent pregnancy	Not in school or paid work	Child marriage
**Predicted probabilities**						
No accelerator	22.3 (16.3 to 28.3)	20.4 (13.3 to 27.5)	13.9 (8.7 to 19)	23.7 (17.7 to 29.7)	28.5 (21.3 to 35.6)	10.0 (5.7 to 14.3)
All three accelerators	5.0 (2.3 to 7.8)	6.0 (1.6 to 10.4)	6.6 (3.4 to 9.7)	7.7 (4.4 to 11.1)	18.4 (9.6 to 27.1)	1.6 (0.4 to 2.8)
**Risk difference**						
All 3 vs. none	-17.2 (-24.3 to -10)	-14.4 (-24.2 to -4.6)	-7.3 (-14.2 to -0.3)	-16.0 (-23.5 to -8.4)	-10.1 (-22.6 to 2.4)	-8.4 (-12.9 to -4)
Gender-equitable attitudes	-7.9 (-12.4 to -3.3)	-5.1 (-10.3 to 0.1)	-1.6 (-5.8 to 2.7)	-5.3 (-9.7 to -0.9)	-4.8 (-10.8 to 1.3)	-2.1 (-5.5 to 1.3)
Food security	-2.6 (-7.3 to 2.1)	-2.8 (-8.8 to 3.3)	-2.2 (-6.8 to 2.4)	-6.2 (-11.3 to -1.2)	-0.8 (-7.6 to 5.9)	-3.4 (-7.3 to 0.5)
High parental monitoring	-8.3 (-13.3 to -3.2)	-7.4 (-13.6 to -1.2)	-3.7 (-7.5 to 0.1)	-4.8 (-9.0 to -0.6)	-4.7 (-12.2 to 2.8)	-3.3 (-5.7 to -0.9)

Note: 95% confidence intervals in parenthesis

## Discussion

There is an urgent need for combination interventions that simultaneously mitigate multiple vulnerabilities for AGYW, such as the ‘triple threat’ of HIV, gender-based violence, and adolescent pregnancy. Our study results suggest that interventions that aim to improve gender-equitable attitudes, parental monitoring and food security may help to reduce violence, adolescent pregnancy, and other factors associated with high HIV exposure risk. Moreover, the combined effects of the protective factors may be greater than individual effects, suggesting the possibility for high impact interventions. Findings build on the emerging evidence on the potential for ‘accelerators’ – interventions, provisions, services, or programmatic areas that simultaneously make positive impact across multiple outcomes [17, 18] – to address multiple vulnerabilities among AGYW.

Our findings indicate that AGYW in Kenya who expressed more positive gender-equitable attitudes were less likely to report IPV and less likely to have had a pregnancy during adolescence. These findings align with studies from both high- and low-income country settings showing that gender-inequitable attitudes are correlated with: experiences of violence among AGYW [35]; higher fertility and lower secondary school completion [36]; lower contraception intentions and lower self-efficacy in condom use [37]. Among men, less equitable gender attitudes are correlated with higher rates of concurrent partners, IPV perpetration, and alcohol abuse, all of which are known to increase the risk of HIV transmission [38]. Inequitable power dynamics are central to harmful gender norms and to limiting contraceptive choice and use, lack of decision-making regarding sex, and adolescents’ lack of control over their own bodies [39, 40].

While the promotion of gender-equitable attitudes holds promise as an accelerator, embedded gender norms are resistant to change and reinforced by the behaviours that children/adolescents witness and experience [40]. Interventions to promote gender-equitable attitudes and norms have generally had mixed to poor effects, and sometimes the only positive impacts have been among men [8, 41, 42]. To transform restrictive gender norms meaningfully, greater investment is needed in the design and evaluation of interventions that address multifactor drivers of gender equality. This will likely require a multi-component approach and intensive engagement with AGYW and their communities.

Our study findings indicate that high parental monitoring is correlated with less IPV, less sexual violence, less adolescent pregnancy and less child marriage among AGYW in Kenya. These findings align with previous accelerator analysis focusing on adolescents living with HIV that showed ​that parenting support was associated with good mental health, no violence perpetration, no high-risk sex, no community violence, and no physical or emotional abuse [13]. Among adolescents in general, positive parenting and high parental monitoring have been associated with less physical and emotional abuse, less HIV-risk behaviour, and less experience of community violence [20]. In addition, the absence of family support has been associated with greater HIV incidence and having multiple sexual partners [27]. Our study builds on the emerging evidence which indicates that parental support can simultaneously act as a development accelerator and have positive impacts on different violence outcomes [14]. As with gender-transformative interventions, the importance of parental monitoring and support in child and adolescent development is clear, but adopting interventions that improve parenting to scale is challenging, given that they are often resource intensive. Moreover, as the level of parental monitoring is heavily influenced by environmental and structural constraints (financial stress, work commitments, unsafe neighbourhoods, poor health and low levels of education), and is rarely a choice, removal of structural barriers is needed to enable parenting interventions to succeed [20, 43].

Our results also align with previous accelerator analyses showing that food security can improve multiple adolescent outcomes in different settings [14, 20, 21]. The direction of the relationship found in our study between food security and both adolescent pregnancy and child marriage is unclear, and potentially multidimensional: e.g., did food insecurity result in behaviours that increase risk of pregnancy [3], or did the extra person to feed in the household reduce food security? Nevertheless, the findings in our study indicate the need for food security interventions for adolescent mothers and AGYW in child marriages to reduce further vulnerability. For example, evidence indicates an inverse relationship between food security and multiple forms of violence [14], and between food security and multiple behaviours that increase the risk of exposure to HIV [3].

Finally, our study identified potential synergistic effects, with the combination of gender-equitable attitudes, high parental monitoring and food security associated with substantially lower risk of violence outcomes, adolescent pregnancy, and other outcomes that increase the risk of exposure to HIV. This finding adds to the mounting evidence that combination programs can offer advantages by maximizing the synergies among components of the program, and specifically that accelerators in combination may have greater effects [13–15, 20, 21, 44]. The challenge remains to identify the most effective combinations of interventions within each setting. This is illustrated by findings showing that the ‘Determined, Resilient, Empowered, AIDS-free, Mentored, and Safe’ (DREAMS) package of interventions did not reduce HIV incidence among AGYW in Kenya [45], but did improve social support and self-efficacy [46]. Another study found that AGYW in South Africa who accessed three or more DREAMS-like interventions were more likely to have tested for HIV and to have accessed contraceptives [47]. While our study found evidence on three potential protective factors, there are many other factors that should be considered. Examples of other factors that may reduce HIV risk, adolescent pregnancy or gender-based violence include: education [27–30]; social protection [8, 48]; formation of partnerships with similar-aged partners [49–52]; and social support [53].

The study’s findings should be interpreted alongside its limitations. The exact timing of the exposure and outcome variables is unknown and limits causal inference between the protective factors and outcomes, as illustrated in the case of food security. In addition, all study measures were self-reported and may be influenced by social desirability bias. Data were not available to disaggregate findings for heterosexual and same-sex relationships. Finally, the overall survey response rate of 74% among females might have introduced sample selection bias.

Overall, our study results show the potential for accelerators to address multiple components of the triple threat of HIV, adolescent pregnancy and gender-based violence among AGYW in Kenya. Moreover, combination interventions that leverage all the identified accelerators – gender-equitable attitudes, food security and parental monitoring – may have the greatest impact. In the context of limited resources and competing priorities, it becomes essential to ensure that investments achieve the highest returns. Multi-component approaches (e.g., integration of gender-transformative content into a parenting programme) are likely to have greater impact and be less expensive than implementing components separately. Further research is needed to understand the most cost-effective combination of interventions [54], and how these can be financed to ensure progress towards achieving the Sustainable Development Goals.

## Electronic Supplementary Material

Below is the link to the electronic supplementary material.

Supplementary Material 1
