# Supplementary material for: Tackling the Triple Threat in Kenya: Factors Associated with Protection against HIV Risk, Gender-Based Violence, and Pregnancy among Adolescent Girls and Young Women
Source: AIDS Behav. 2025 Feb 13;29(6):1738–46. doi: 10.1007/s10461-025-04643-9 (PMC12075351; doi:10.1007/s10461-025-04643-9)
Supplement: Supplementary file 1 — Supplementary Material 1 [file 10461_2025_4643_MOESM1_ESM.docx]

**Supplemental Material File 1**

**Article**: Tackling the Triple Threat in Kenya: Factors associated with protection against HIV risk, gender-based violence, and pregnancy among adolescent girls and young women

**Table S1.** Multivariate regression models of the associations between hypothesised accelerators and outcomes

|  | Intimate partner violence | |  | Sexual violence | |  | High HIV exposure risk | |  | Adolescent pregnancy | |  | Not in school or paid work | |  | Child marriage | |
| --- | --- | --- | --- | --- | --- | --- | --- | --- | --- | --- | --- | --- | --- | --- | --- | --- | --- |
|  | aOR (95% CI) | p |  | aOR (95% CI) | p |  | aOR (95% CI) | p |  | aOR (95% CI) | p |  | aOR (95% CI) | p |  | aOR (95% CI) | p |
| Hypothesised accelerators |  |  |  |  |  |  |  |  |  |  |  |  |  |  |  |  |  |
| Gender-equitable attitudes | 0.47 (0.28–0.78) | 0.004 |  | 0.63 (0.38–1.06) | 0.083 |  | 0.82 (0.48–1.41) | 0.476 |  | 0.58 (0.36–0.94) | 0.028 |  | 0.72 (0.47–1.11) | 0.139 |  | 0.61 (0.24–1.51) | 0.280 |
| Food security | 0.80 (0.54–1.19) | 0.267 |  | 0.80 (0.49–1.29) | 0.353 |  | 0.78 (0.46–1.30) | 0.332 |  | 0.57 (0.37–0.88) | 0.012 |  | 0.94 (0.60–1.48) | 0.803 |  | 0.51 (0.26–1.00) | 0.049 |
| Parental support | 0.44 (0.25–0.76) | 0.004 |  | 0.49 (0.25–0.98) | 0.044 |  | 0.62 (0.36–1.04) | 0.071 |  | 0.61 (0.38–0.97) | 0.038 |  | 0.72 (0.41–1.26) | 0.248 |  | 0.41 (0.20–0.83) | 0.014 |
| Covariates |  |  |  |  |  |  |  |  |  |  |  |  |  |  |  |  |  |
| Rural residence | 0.75 (0.43–1.30) | 0.301 |  | 0.96 (0.52–1.76) | 0.891 |  | 0.67 (0.40–1.12) | 0.125 |  | 0.80 (0.48–1.32) | 0.375 |  | 1.00 (0.58–1.73) | 1.000 |  | 0.54 (0.27–1.09) | 0.084 |
| Lived in poor household | 1.45 (0.83–2.51) | 0.190 |  | 0.97 (0.54–1.73) | 0.910 |  | 1.94 (1.08–3.50) | 0.027 |  | 3.00 (1.74–5.15) | <0.01 |  | 1.35 (0.86–2.12) | 0.194 |  | 2.02 (0.90–4.53) | 0.088 |
| Age (in years) | 1.27 (1.18–1.36) | <0.01 |  | 0.96 (0.90–1.01) | 0.143 |  | 1.42 (1.32–1.52) | <0.01 |  | 1.45 (1.35–1.56) | <0.01 |  | 1.19 (1.11–1.27) | <0.01 |  | 1.31 (1.20–1.43) | <0.01 |
| Absent parent | 1.38 (0.79–2.44) | 0.260 |  | 1.12 (0.65–1.92) | 0.683 |  | 1.02 (0.64–1.63) | 0.933 |  | 1.97 (1.32–2.94) | <0.01 |  | 0.67 (0.44–1.00) | 0.050 |  | 1.69 (0.87–3.26) | 0.118 |
| Secondary school completed | 1.02 (0.60–1.73) | 0.949 |  | 1.06 (0.60–1.87) | 0.840 |  | 0.93 (0.57–1.51) | 0.762 |  | 0.55 (0.30–1.01) | 0.053 |  | 5.24 (3.03–9.09) | <0.01 |  | 0.30 (0.14–0.67) | 0.003 |
|  |  |  |  |  |  |  |  |  |  |  |  |  |  |  |  |  |  |
| N | 1301 |  |  | 1301 |  |  | 1301 |  |  | 1301 |  |  | 1301 |  |  | 1301 |  |


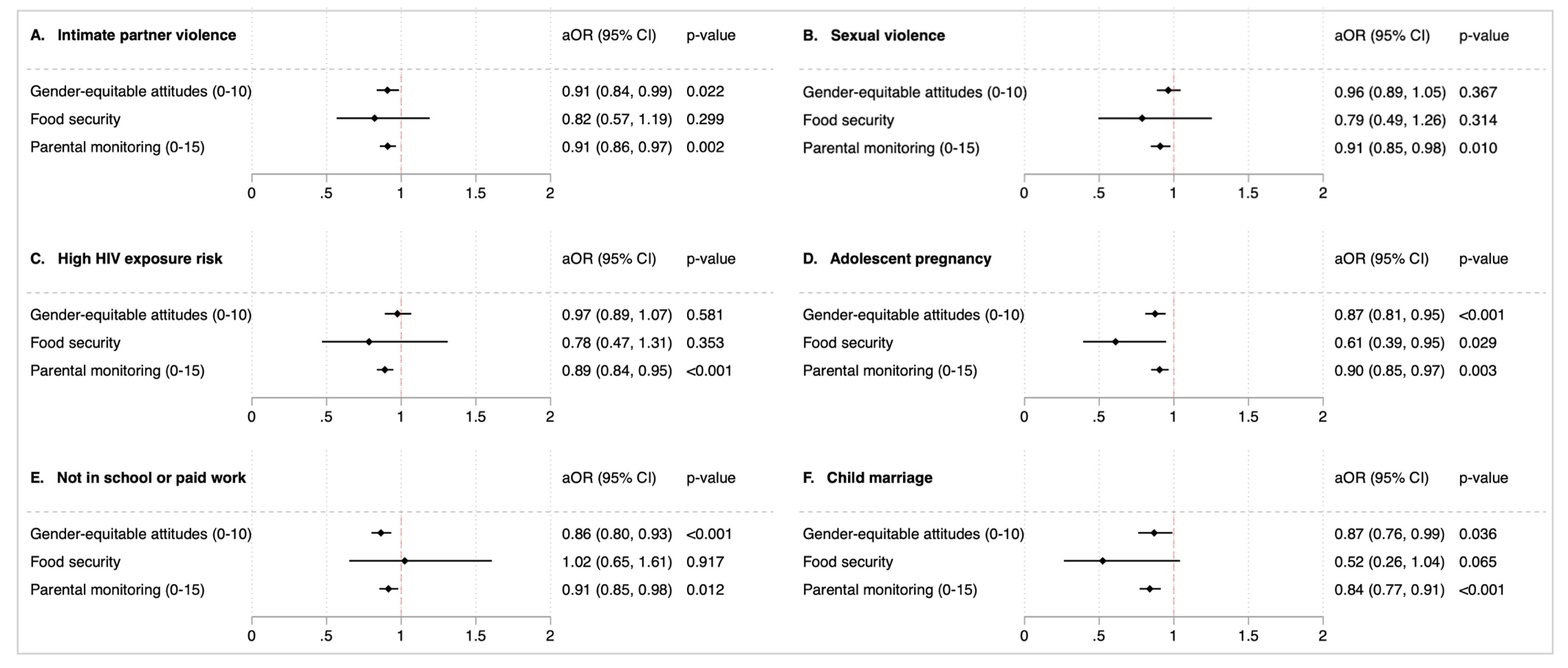


**Figure S1**. Associations between hypothesised accelerators and individual outcomes: Figure S1A. Intimate partner violence; Figure S1B. Sexual violence; Figure S1C. High HIV exposure risk; Figure S1D. Adolescent pregnancy; Figure S1E. Not in school or paid work; Figure S1F. Child marriage. Adjusted odds ratios (aOR) and 95% confidence intervals (CI) are adjusted for rural (vs. urban) residence, age, household poverty, and absent parent, and secondary school completion.
